# Crystal Structures of Group B Streptococcus Glyceraldehyde-3-Phosphate Dehydrogenase: Apo-Form, Binary and Ternary Complexes

**DOI:** 10.1371/journal.pone.0165917

**Published:** 2016-11-22

**Authors:** Norbert Schormann, Chapelle A. Ayres, Alexandra Fry, Todd J. Green, Surajit Banerjee, Glen C. Ulett, Debasish Chattopadhyay

**Affiliations:** 1 Department of Medicine, University of Alabama at Birmingham, Birmingham, Alabama 35294, United States of America; 2 Department of Microbiology, University of Alabama at Birmingham, Birmingham, Alabama 35294, United States of America; 3 North-Eastern Collaborative Access Team and Department of Chemistry and Chemical Biology, Cornell University, Argonne, Illinois 60439, United States of America; 4 School of Medical Science, and Menzies Health Institute Queensland, Griffith University, Parklands 4222, Australia; NCI at Frederick, UNITED STATES

## Abstract

Glyceraldehyde 3-phosphate dehydrogenase or GAPDH is an evolutionarily conserved glycolytic enzyme. It catalyzes the two step oxidative phosphorylation of D-glyceraldehyde 3-phosphate into 1,3-bisphosphoglycerate using inorganic phosphate and NAD^+^ as cofactor. GAPDH of Group B Streptococcus is a major virulence factor and a potential vaccine candidate. Moreover, since GAPDH activity is essential for bacterial growth it may serve as a possible drug target. Crystal structures of Group B Streptococcus GAPDH in the apo-form, two different binary complexes and the ternary complex are described here. The two binary complexes contained NAD^+^ bound to 2 (mixed-holo) or 4 (holo) subunits of the tetrameric protein. The structure of the mixed-holo complex reveals the effects of NAD^+^ binding on the conformation of the protein. In the ternary complex, the phosphate group of the substrate was bound to the new Pi site in all four subunits. Comparison with the structure of human GAPDH showed several differences near the adenosyl binding pocket in Group B Streptococcus GAPDH. The structures also reveal at least three surface-exposed areas that differ in amino acid sequence compared to the corresponding areas of human GAPDH.

## Introduction

Glyceraldehyde 3-phosphate dehydrogenase (GAPDH; EC 1.2.1.12) is an essential enzyme conserved in all species. GAPDH plays a key role in glycolysis and gluconeogenesis by catalyzing the reversible oxidative phosphorylation of D-glyceraldehyde 3-phosphate (D-G3H) to the energy-rich intermediate glyceraldehyde 1,3-bisphosphate (1,3-BPG). In addition, GAPDH is increasingly recognized to exhibit a wide range of biological functions [1–2]. Extracellular GAPDHs have been reported to be involved in pathogenesis of many bacteria [3–5]. Prominent among them is the surface-associated GAPDH protein of *Streptococcus agalactiae* or Group B Streptococcus (GBS). GBS is a leading cause of infections in newborns, pregnant women and older persons with chronic illness. It is also the most common cause of infection of the blood (septicemia) and of the brain (meningitis) in newborns. Recent studies suggest that GBS GAPDH is a major virulence factor [6–7] and a potential vaccine candidate [8–9]. Immunization of pregnant mice with recombinant GBS GAPDH conferred antibody-mediated protection to newborns against infection with highly virulent strains of GBS [8]. However, developing a GAPDH-based vaccine may be challenging because the sequences and structures of GAPDHs across the species are very similar. It is imperative that the antibodies generated by the vaccine do not cross-react with human GAPDH (hGAPDH). Thus a comparative structure-function analysis of GBS GAPDH and hGAPDH would be highly important for designing a safe vaccine antigen. Previously, we determined the crystal structure of GBS GAPDH in the holo-form at 2.46 Å resolution. Interestingly, this structure revealed a novel surface area, which is not present in hGAPDH [10]. Although the functional implication of this distinct feature is not known at this time, this finding underscored the need for a detailed analysis of the GBS GAPDH structure. As an essential enzyme for the survival of GBS, GAPDH may be a potential target for developing antibacterial drugs. Therefore, crystal structures of the enzyme with substrate/product or analogs bound are necessary for identifying any novel binding-pockets for selective inhibitors. Moreover, among the 104 entries for GAPDH crystal structures that have been deposited in the Protein Data Bank, substrate/product- or substrate-analog-bound structures are available only for three enzymes. Structures of ternary complexes are available only for GAPDHs from *Bacillus stearothermophilus* (*Bs*GAPDH) [11–12], *Staphylococcus aureus* (*Sa*GAPDH) [13] and *Cryptosporidium parvum* (*Cp*GAPDH) [14]. To capture the enzyme in the substrate-bound state in most cases the active site cysteine was mutated to serine, alanine or glycine. These structures reveal variations in the phosphate binding sites in GAPDH and suggest alternative binding modes or movement of substrate molecules during the reaction, and emphasize the significance of additional structural studies on complexes representing different enzymatic states of GAPDHs.

### Reaction Mechanism

GAPDH-catalyzed phosphorylation of D-G3H takes place in two steps. In the first exergonic reaction the aldehyde group of D-G3H is converted into a carboxylic acid with concomitant reduction of NAD^+^ to NADH. The energy released by this reaction drives the endergonic second reaction in which a molecule of inorganic phosphate is transferred to the intermediate acid to form the product 1,3-BPG. The reaction mechanism involves formation of a covalent bond between the thiol group of a conserved cysteine residue of GAPDH and the carbonyl C-atom of D-G3H resulting in the formation of the hemithioacetal intermediate. A hydride ion is transferred from D-G3H to the cofactor NAD^+^ to form NADH while oxidation of D-G3H by a water molecule generates a thioester intermediate. In the second step, the thioester is phosphorylated in a nucleophilic attack by an inorganic phosphate (P_i_) ion resulting in the formation of the product 1,3-BPG and the release of the thiol-group of the active site cysteine.

Originally, two sites where sulfate ions were located in *Bs*GAPDH crystal structures were designated as ‘Ps’ and ‘Pi’ sites for binding of the phosphate group of the substrate (D-G3H) and the inorganic phosphate ion, respectively [15]. Subsequently, in crystals of *Leishmania mexicana* GAPDH (*Lm*GAPDH) grown in phosphate buffer a second ‘Pi’ site was identified approximately 2.9 Å away from the original ‘Pi’ site and was named the ‘new Pi’ site [16]. According to the flip-flop reaction mechanism, the C-3 phosphate group of the substrate initially binds to the ‘Pi’ site in the acylation step and then flips to the ‘Ps’ site during the phosphorylation step as suggested by Skarzyński *et al*. [15]. However, in the thioacyl intermediate of the wild-type enzyme, the C3-phosphate occupied the ‘new Pi’ site in all four subunits of the tetramer. In the *Cp*GAPDH active site serine mutant the C3-phosphate of D-G3H was found at the ‘new Pi’ site in three subunits, while in the fourth subunit the phosphate was bound to a novel site. Moreover, the conformation of the substrate in different subunits varied [14]. The C3-phosphate has also been located in the ‘new Pi’ site in *Sa*GAPDH [13] thereby suggesting that the ‘new Pi’ site is the preferred binding site. On the other hand, in the ternary complexes of the active site *Bs*GAPDH mutants, the C-3 phosphate of the non-covalently bound D-G3H was located in the ‘Ps’ site [11]. These structures indicate a considerable flexibility in the active site in this highly conserved enzyme and emphasize the need for further structural investigation.

Here we present three new crystal structures of GBS GAPDH: an apo-form; a mixed apo/holo-state, and a ternary complex. In the mixed apo/holo-form (referred to as mixed-holo) NAD^+^ is bound in two subunits while the other two subunits are in the apo-state. The ternary complex contains NAD^+^ and D-G3H bound to the C152S mutant. In addition, we have determined the structure in the holo-form using a new data set extending to a resolution of 2.0 Å. This structure supersedes our previously determined structure [PDBID: *4QX6*], which was refined to 2.46 Å resolution. We also provide a comparison of the enzyme active site in GBS GAPDH and hGAPDH.

## Results and Discussion

### Overall Structure

**Fig 1:**
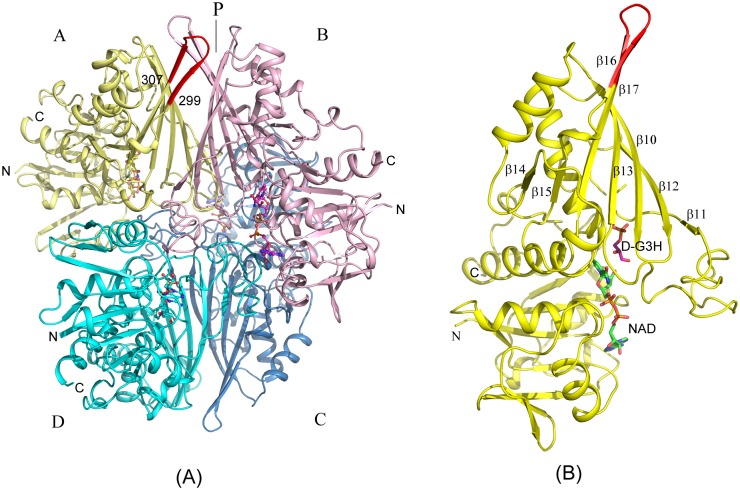
GBS GAPDH Assembly. (A)Tetrameric assembly of GBS GAPDH. Subunits A (yellow), B (light magenta), C (sky blue) and D (Cyan) of the holo complex (*5JY6*) are shown in cartoon representation. NAD^+^ bound to each subunit is shown in ball and stick model. N and C-terminii of A, B and D subunits are labeled. The extended β-sheet (residues 299–307) in the A subunit is colored red. The major interface (P) in the tetramer is indicated. (B) Cartoon drawing showing GBS GAPDH monomer A subunit. Beta-strands at the P-interface in the tetramer, and the substrate and the cofactor are labeled.

The GBS GAPDH apo structure crystallized in space group P2_1_2_1_2_1_ and crystals of the holo, mixed-holo and ternary complexes belong to space group P2_1_ (Table 1). GBS GAPDH exists as a tetramer. In the crystal structures the subunits (A, B, C and D) in the asymmetric unit are related by 222 non-crystallographic (NCS) symmetry (Fig 1). The overall structure and topology of GBS GAPDH are similar to other GAPDHs [10–21]. Each GAPDH subunit is composed of two domains, and consists of 13 helices and 2 β-sheets of 9 and 8 strands. In the holo enzyme all four subunits have NAD^+^ bound in the active site while in the mixed-holo complex only two subunits contain NAD^+^. All subunits in the ternary complex contain NAD^+^ and substrate D-G3H. The overall quality of the structures is excellent; all residues in the structures of the mixed-holo, holo and ternary complexes are in the allowed regions of the Ramachandran plot, and only the tetramer of the apo-form contains 8 residues in the disallowed region. In subunits A, B and D of the apo-enzyme structure several residues in the vicinity of the cofactor binding pocket are disordered and could not be modelled in the electron density. For all four structures the average B-factors are close to the estimated Wilson B-factor, and Molprobity scores are also good (Table 1). Average B-factors for ligands are generally lower than for protein residues. The quality of electron density for cofactor and substrate is good (see S1, S2 and S3 Figs; [NAD^+^: 2mFo-DFc map at 1σ contour level, S1 and S2 Figs; D-G3H: 2mFo-DFc omit map at 3σ contour level, S3 Fig].

**Table 1:** Data collection and refinement statistics for the four structures of GBS GAPDH.

Crystal	Apo	Apo/Holo	Holo	Ternary
	(*5JYF*)	(*5JYE*)	(*5JY6*)	(*5JYA*)
***Data collection***				
Space Group	P2_1_2_1_2_1_	P2_1_	P2_1_	P2_1_
a, b, c [Å]	79.22, 112.63, 147.63	78.25, 107.36, 87.90	67.58, 104.47, 89.27	68.03, 108.68, 90.99
β [°]		113.0	104.1	105.8
No. Molecules in ASU^1^	4	4	4	4
Resolution [Å]	147.63–2.62 (2.76–2.62)^2^	107.36–2.23 (2.29–2.23)	86.60–2.00 (2.11–2.00)	30.00–2.85 (2.90–2.85)
Unique reflections	40180 (5805)	64548 (4531)	82890 (12103)	29605 (1444)
Completeness	99.5 (99.9)	99.1 (98.8)	99.3 (99.1)	99.7 (99.7)
Multiplicity	4.1 (4.1)	3.4 (3.4)	2.5 (2.5)	3.7 (3.7)
R_merge_ [%]	8.6 (84.0)	4.0 (80.3)	8.4 (46.7)	5.7 (24.1)
I/σ (I)	9.7 (1.1)	13.4 (1.1)	11.3 (1.9)	9.6
***Refinement***				
Resolution [Å]	89.55–2.62 (2.69–2.62)	80.91–2.23 (2.29–2.23)	86.60–2.00 (2.05–2.00)	87.55–2.85 (2.92–2.85)
No. of reflections	40097 (2912)	64504 (4738)	82851 (6118)	29579 (1959)
Completeness [%]	99.2 (99.2)	98.9 (98.4)	99.2 (98.7)	98.8 (89.7)
R_work_ [%]	23.12 (39.7)	20.74 (39.4)	19.49 (29.8)	17.42 (26.0)
R_free_^3^ [%]	27.84 (39.1)	24.87 (43.0)	21.63 (30.7)	21.16 (30.0)
Wilson B [Å^2^]	52.6	52.6	24.5	42.6
**Average B-factors [Å**^**2**^**]**				
Overall	60.3	60.8	29.7	43.1
Protein/Ligands/Water	60.5/-/37.9	61.1/49.2/50.2	29.7/23.2/30.5	43.5/39.6/26.6
No of solvent molecules	91	151	520	161
V_M_^4^ (% solvent)	2.1 (40%)	2.1 (42%)	2.0 (37%)	2.0 (39%)
Map CC (Fc, 2mFo-DFc)	0.77	0.76	0.84	0.85
CC (Fo-Fc)(Fo-Fc free)	0.93/0.89	0.96/0.94	0.95/0.94	0.94/0.91
*rmsd* bonds [Å]/ angles [°]	0.008	0.012	0.014	0.014
Ramachandran (core)	93.2%	95.3%	96.5%	95.1%
Clash score	2.15	1.83	0.54	0.54
MolProbity score	1.43	1.27	0.91	1.03

^1^Asymmetric Unit;

^2^numbers in parenthesis are for highest resolution shell;

^3^test set uses~5% data;

^4^Matthews Coefficient

### Assembly, Interfaces and Interactions of GBS GAPDH Subunits

Like other tetrameric phosphorylating GAPDHs, the GBS GAPDH tetramer is assembled as a dimer of dimers and displays three non-equivalent interfaces designated P, R and Q. These interfaces have been studied in detail for their involvement in cooperativity, which refers to the transmission of conformational changes across subunit interfaces and the concomitant effects on ligand-binding. For example, GAPDH from yeast exhibited positive cooperativity for NAD^+^ binding as binding of NAD^+^ to one subunit resulted in an increase in the affinity in another subunit. On the other hand, mammalian GAPDH and *Bs*GAPDH showed negative cooperativity. Roitel *et al* [18] showed that the P interface is involved in the cooperative binding of NAD^+^ in *Bs*GAPDH.

**Fig 2:**
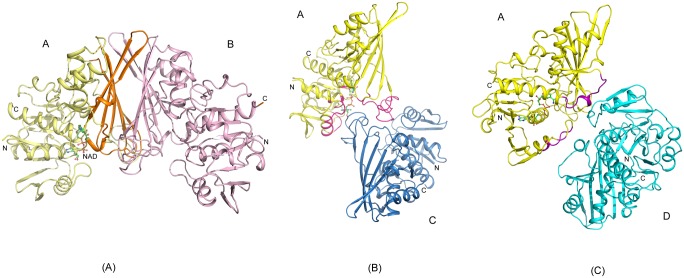
Dimer Interfaces in GBS GAPDH. The subunits of the holo complex [*5JY6*] forming the dimer interfaces are shown in cartoon representation. Subunits are colored A (yellow), B (light magenta), C (sky blue) and D (cyan). (A) P Interface: Subunits A and B forming the P interface are displayed. The interface areas on A are colored in orange. (B) R Interface: Subunits A and C at the R interface are shown. The interface regions on A are in hot pink color. (C) Q Interface: Subunits A and D are shown. The Q interface areas on A are shown in purple color. The P interface is the most extensive in all four structures followed by the R and the Q interface.

The dimers in GBS GAPDH tetramer are composed of subunit pairs A, B and C, D. As shown in Figs 1A and 2A, subunits of the dimers form the major interface P [18]. The P interface is formed between β-strands 10–17 in the catalytic C-terminal domain (residue assignment based on holo enzyme; strand 10: 170–180; strand 11: 208–210; strand 12: 229–236; strand 13: 242–250; strand 14: 271–274; strand 15: 290–293; strand 16: 298–302; strand 17: 305–314) and shows the most extended surface contacts (area: 3795–3893 Å^2^ in different dimers; see S1 and S2 Tables).

The second largest interface, the R interface, includes residues in the N-terminal domain that interact with NAD^+^ and loop residues 181–206 in the C-terminal domain of subunit pairs A, C and B, D (area: 2518–2790 Å^2^; Fig 2B; see also S1 and S2 Tables). The smallest interface, the Q interface, shows limited interactions between residues in the ranges 43–53 and 274–291 of adjacent subunits A, D and B, C (area: 997–1093 Å^2^; Fig 2C; S1 and S2 Tables). The surface areas for the P and Q interfaces are similar in the four structures presented here. The area for the R interfaces increases in the order ternary > holo > mixed-holo > apo indicating additional interactions between subunits across this interface upon cofactor and substrate binding. Overall root mean square deviations (*rmsd*) between individual subunits in the structure of the apo-form are in the range of 0.42–0.99 Å (S3 Table). In the mixed-holo complex NAD^+^ was bound in one subunit of each dimer- subunit A of the AB dimer and subunit C of the CD dimer contained NAD^+^, while B and D subunits remained in the apo-state. Presence of a mixed-holo form was observed previously in the crystal structure of GAPDH from rabbit muscle [21]. In the mixed-holo structure electron density was sufficiently continuous for fitting all residues in each subunit including the apo-subunits. This structure further illustrates the effects of conformational changes induced by NAD^+^ binding in the individual subunits. The *rmsd* between subunits with and without NAD^+^ are 1.2–1.3 Å. In comparison, the *rmsd* between the pairs of apo-subunits (0.33 Å) or holo-subunits (0.2 Å) in this structure is much lower. In the holo-enzyme and the ternary complex each subunit contains a bound NAD^+^ molecule and *rmsd* between the individual subunits are in the range of 0.06–0.14 Å and 0.09–0.26 Å, respectively (S3 Table). However, electron density for several residues was also missing from the protein chains in the holo-enzyme structure and in the ternary complex. It should be noted that the holo-enzyme structure described here [*5JY6*] is based on a new data set extending to 2.0 Å resolution. Based on the refinement parameters (R and R_free_, and average B-factors), and the model quality judged by Molprobity scores [22] and Ramachandran plot, this structure represents an improved version of the previously reported structure [*4QX6*].

### Cofactor binding

**Fig 3:**
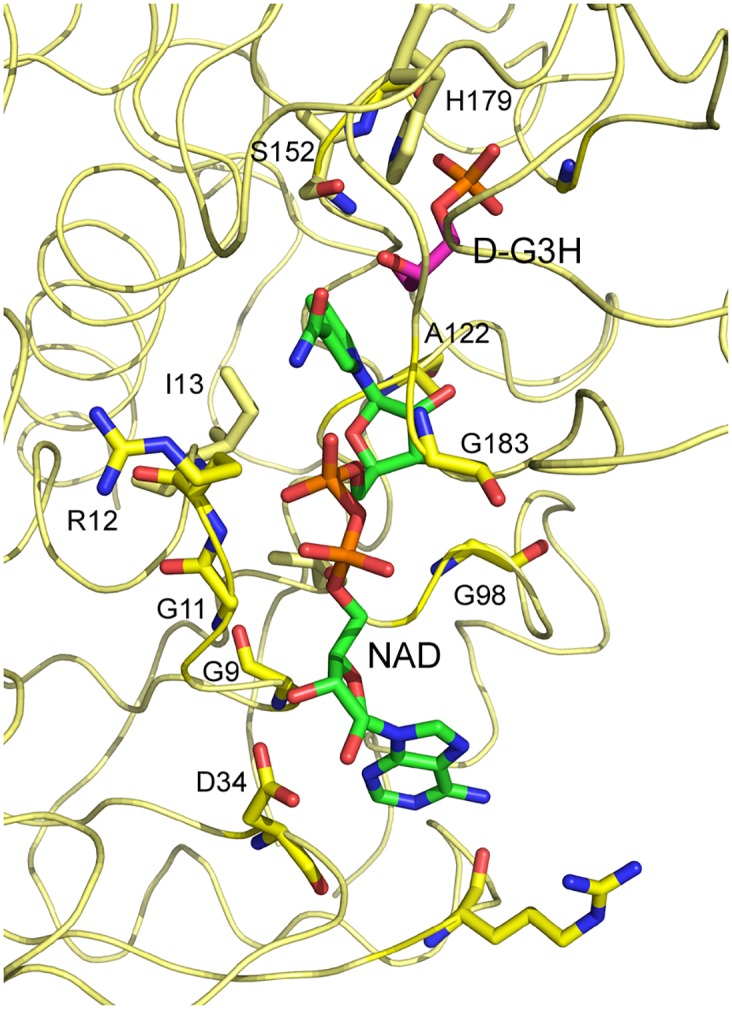
Close-up View of the GBS GAPDH Active Site. NAD^+^, D-G3H and residues within 3.5 Å from either ligand are displayed as stick models. Color code: N blue, O red, P orange and C yellow (protein), green (NAD^+^), magenta (D-G3H)

The NAD^+^-binding site in GAPDH is located in the N-terminal domain, which exhibits the typical Rossmann-fold. The NAD^+^-binding site and the interacting active site residues in GBS GAPDH are shown in Fig 3. The NAD^+^ molecule binds to residues 8–15, 34–35, 78, 96–98, 121, 152, 316 and 320. Of these residues, residues 9–15 (-GFGRIGR-), 34 (-D-), 97–98 (-TG-), 152 (-C-), 316 (-N-) and 320 (-Y-) are strictly conserved in human, bacterial and parasitic GAPDHs. The NAD^+^ pyrophosphate moiety binds to the glycine-rich loop (residues 9–15) with hydrogen bonds provided by backbone amino groups of Arg12 and Ile13 (Fig 3). The nicotinamide carbonyl oxygen is hydrogen bonded to Asn316 (3.0 Å distance between ND2 and O7N). In addition, Asp34 forms two hydrogen bonds with the adenosine ribose. Two conserved water molecules bridge the cofactor pyrophosphate and the glycine-rich loop in the active site of all four subunits in the holo complex as described for hGAPDH [19].

Previously, we reported that NAD^+^-binding in *Cp*GAPDH resulted in the stabilization of a loop called S-loop [14]. In the apo-*Cp*GAPDH structure [*1VSU*] this loop was completely disordered while in the holo-form the entire loop could be fitted in the electron density [*1VSV*]. In GBS GAPDH, the S-loop corresponds to residues 186–192. In the GBS GAPDH apo-state all residues in this loop were ordered in each subunit but it represented one of the most divergent areas in the tetramer.

The active site residue Cys152 is positioned between the nicotinamide moiety of NAD^+^ and the side chain of active site residue His179. In the apo-enzyme structure the distance between Cys152 SG and His179 NE2 in different subunits varies in the range 4.0–4.2 Å. In the holo-form, the distance is 3.4–3.5 Å. The difference in distance between Cys152 SG and His179 NE arises from the movement of the side chains of both residues upon NAD^+^ binding. Thus in the mixed-holo complex, the corresponding distance in the NAD^+^-bound subunits (A and C) is 3.5 Å while the distances are 3.8 and 4.0 Å in the other two subunits (NAD-free-subunits). Modeling of a cysteine residue in place of Ser152 in the structure of the ternary complex shows that the SG atom remains at a similar distance (3.6 Å) from His179 NE2 in all four subunits. Details of hydrogen bonds involving cofactor and substrate are presented in S4 Table.

### Substrate Binding

**Fig 4:**
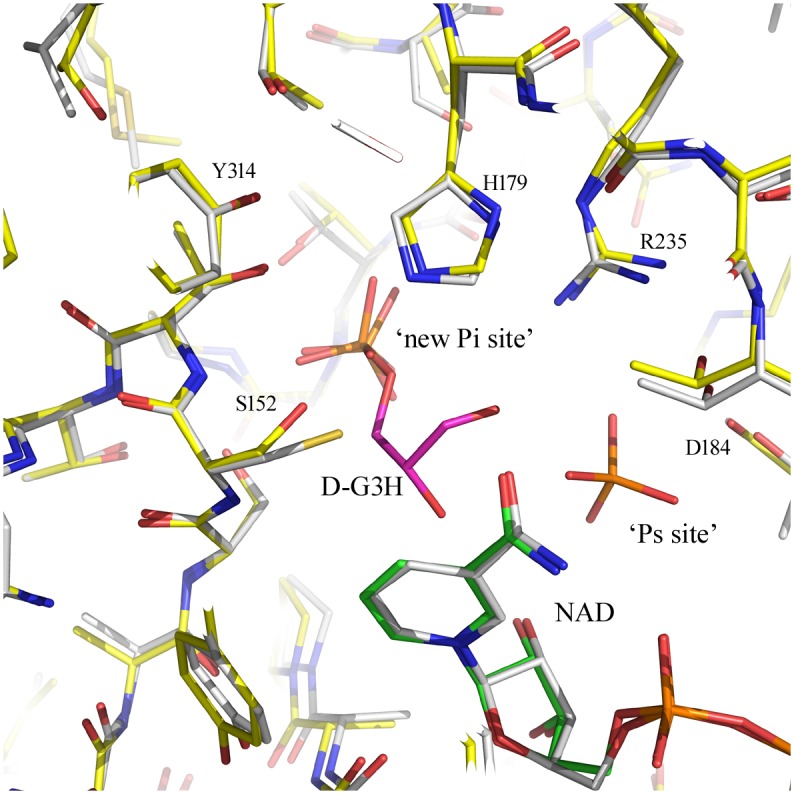
Superimposition of GBS GAPDH Ternary Complex [*5JYA*] and *Lm*GAPDH Holo Complex [*1A7K*]. The figure presents the superimposition of active site residues in subunit A of GBS GAPDH ternary complex [*5JYA*] and *Lm*GAPDH holo complex [*1A7K*]. Protein residues and ligands are shown as stick models. Color code; O red, N blue, P orange in all; *5JYA*: C atoms yellow (protein), green (NAD^+^) magenta (D-G3H); *1A7K*: C atoms white (protein), white (NAD^+^), inorganic phosphate ions in *A7K* are also shown in stick model. The phosphate group of D-G3H binds is located the ‘new Pi’ site and not the ‘Ps’ site.

We prepared the ternary complex of GBS GAPDH by co-crystallization of an active site mutant C152S enzyme with NAD^+^ and the substrate D-G3H. In GAPDH structures a phosphate ion or the phosphate group of the substrate are located in three different sites, the ‘Pi’ site, the ‘new Pi’ site, and the ‘Ps’ site. In the present complex, the C3-phosphate of the substrate binds at the ‘new Pi’ site in all four subunits. The positions of the substrate were verified based on the 2mFo-DFc map, and the mFo-DFc omit map contoured at 3.0 σ (S2 and S3 Figs). When superimposed onto our ternary complex the position of the phosphate ion in the *E*. *coli* GAPDH structure [*3L6O*] and the position of one of the two phosphate ions in the *Lm*GAPDH structure [*1A7K*] coincide with the placement of the substrate D-G3H phosphate group in the ‘new Pi’ site (Fig 4). Interestingly, based on this superimposition the second phosphate ion in *Lm*GAPDH occupies a position on the opposite side of the substrate (2.3 Å distance from carbonyl oxygen atom O1) that constitutes the ‘Ps’ site, and in *Bs*GAPDH ternary complexes the phosphate group of the substrate D-G3H is bound here [11]. The phosphate group of the substrate forms hydrogen bonds with Ser151 OG, Thr153 OG, Gly213 (backbone N atom) and Thr212 OG. There is some difference in the orientation of the glyceraldehyde moiety in different subunits. In subunits B and D, a rotation around the C3-O1P bond allows the formation of a hydrogen bond between Ser152 OG and the hydroxyl oxygen O2 of the substrate. The distance between Ser152 OG and the O2 atom of the substrate is 2.6 and 3.1 Å in subunits B and D, respectively. In subunit C, the corresponding distance is 3.7 Å. In subunit A, the substrate is rotated in a way that the O2 atom points away from Ser152 OG. In this subunit, Ser152 OG is at a distance of 3.2 Å from the carbonyl C1 atom of the substrate. Both catalytic residues (Ser152 and His179) superimpose well in all subunits and the distance between Ser152 OG and His179 NE is ~3.8 Å. Superimposition of holo-enzyme and ternary complex reveals that the position of His179 is identical in both structures. In addition, no movement of cofactor NAD^+^ is observed. One difference between holo-enzyme and ternary complex are the observed interactions of Arg235. In the holo-enzyme Arg235 forms a salt bridge with Asp184 and hydrogen bonds with the side chains of Thr182 and Gln185. In the ternary complex the residue moves closer to the ‘new Pi’ site where the substrate phosphate group is bound (4.6 Å distance), which leads to disruption of the salt bridge with Asp184 while hydrogen bonds with side chains of Thr182 and Gln185 remain.

### Structural movements upon cofactor and substrate binding

**Fig 5:**
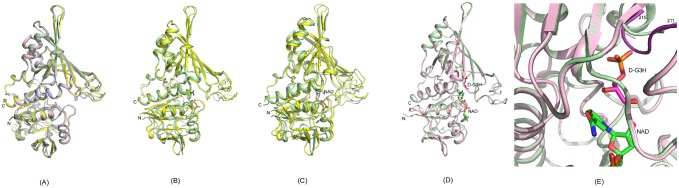
Conformational changes in GBS GAPDH Structures. (A) Cartoon diagram showing superposition of four subunits of the apo-form. (B) Cartoon drawing shows superposition of structures of the A subunit from the apo-form (yellow) with B-subunit of the mixed holo form (light green). The C-subunit of the mixed holo form contains bound NAD^+^ shown in stick model. (C) Structure of the A subunit of the apo-form (yellow) and the A subunit of the holo-complex (pale green) are superposed. NAD^+^ bound in the holo-form is shown in stick model. (D) Cartoon diagram displaying superposition of the structures of the B subunit of the holo form (light green) and the D-subunit of the ternary complex (light magenta). NAD^+^ and D-G3H in the ternary complex are shown in stick models and labeled. (E) A close up view of the diagram in (D) showing the movement of the loop residues 211–215 (labeled) in the ternary complex (in dark purple) towards the phosphate group of the substrate.

Upon cofactor binding GAPDH undergoes substantial conformational changes. These changes lead to stabilization of the protein structure as is reflected in a significantly lower average B-factor for the holo-complex (29.7 Å^2^) as compared to the apo-form (60 Å^2^) and the mixed-holo form (60.8 Å^2^). In the holo complex 45% of the accessible surface area is buried in comparison to 33% in the apo structure (S2 Table). The average B-factor for the ternary complex is 43.1 Å^2^. The effect of NAD^+^ binding was most pronounced in the conformational variations among the two sets of subunits in the mixed-holo complex. The *rmsd* values for superposition of NAD^+^-bound subunits and apo-subunits are 1.23–1.36 Å while the NAD^+^-bound subunits (A and C), and the NAD^+^-free subunits B and D superimpose well with each other. Fig 5 shows superimposition of GBS GAPDH structures in different states.

Substrate binding to the holo enzyme induces much smaller changes (Fig 5D). The main difference is the movement of loop 211–215 that moves towards the phosphate group of the substrate in the ‘new Pi’ site (Fig 5E).

### Comparison with human GAPDH

**Fig 6:**
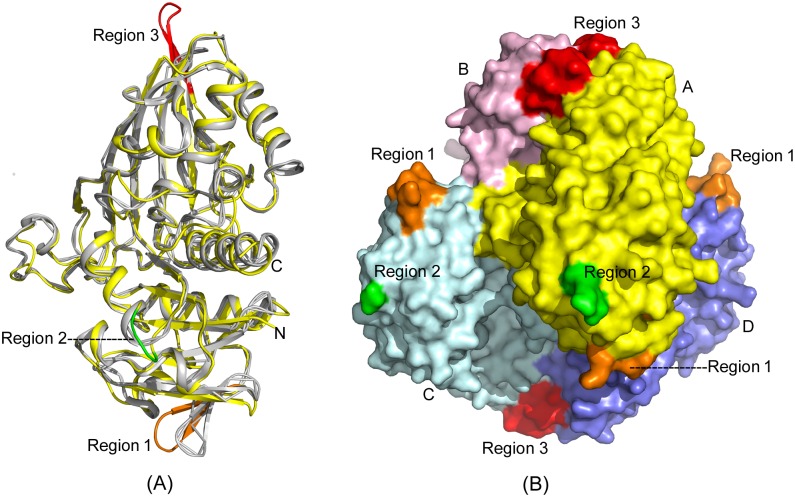
Comparison with human GAPDH. Structures of GBS GAPDH holo-state [*5JY6*] and human GAPDH holo-form [*1U8F*] were superimposed. (A) Protein chains are shown in yellow (GBS GAPDH) and grey (hGAPDH). Three regions in GBS GAPDH corresponding to residues 59–67 (Region1, orange), 111–113 (Region 2, green) and 299–307 (Region 3, red) were found to be the most divergent. (B) The three regions 1, 2 and 3 shown in respective colors on the GBS GAPDH tetramer surface. GBS GAPDH subunit A is in the same orientation as in (A). Subunits are colors are same as in Fig 1 left panel.

**Fig 7:**
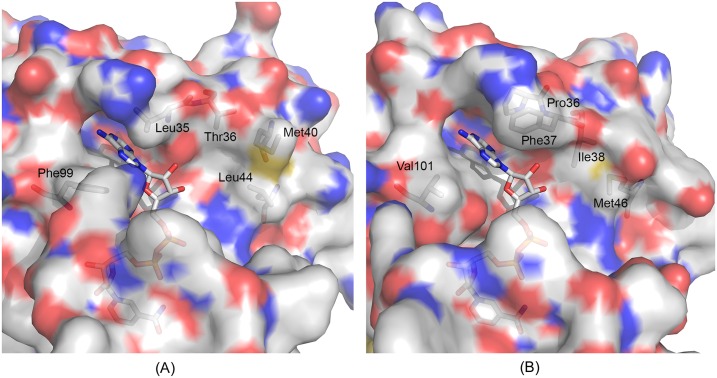
Adenosyl binding pocket in GAPDH. Comparison of the adenosyl moiety in the NAD^+^ binding pocket for (A) GBS GAPDH [*5JY6*], (B) human GAPDH [*1U8F*]. NAD^+^ is shown in stick model. Residues that are different in human and GBS GAPDH are labeled. All figures were prepared with PyMol [36].

GAPDH is an evolutionarily conserved enzyme. The amino acid sequences of GAPDHs from different organisms are similar. The high degree of homology in the sequences is reflected in the similarity in their three-dimensional structures. Comparison of the structures of GBS GAPDH with hGAPDH revealed maximum divergence in three areas corresponding to residues 59–67 (region 1), 111–113 (region 2) and 299–307 (region 3). (see S5 Table for sequence alignment). These areas are shown in orange, green and red respectively in Fig 6. Among these, region 3 displays an extension of a β-strand in GBS GAPDH. It also represents the area of most divergent amino acid sequence in GAPDHs. We have previously discussed the structural characteristic of this region [10]. In region 1, which is comprised of nine residues, hGAPDH and GBS GAPDH share only one identical residue (Gly63 in GBS) and two conservative substitutions (S5 Table). The short loop region 2 is one residue shorter in hGAPDH and the sequences in GBS GAPDH and hGAPDH are His-Glu-Asn and Gln-Gly in hGAPDH, respectively. These three areas remain surface exposed on the GBS GAPDH tetramer. Therefore, antibodies targeting these regions may be able to discriminate between the bacterial protein and the human counterpart. These areas thus may be of interest for vaccine design programs. On the other hand, the catalytic residues and those at the active sites exhibit a high degree of identity in different GAPDHs. Nonetheless, due to their essential role in cellular metabolism GAPDHs are considered potential drug targets. In particular, inhibitors of GAPDH exhibiting antiparasitic activity against trypanosomatid parasites have been developed [23]. For designing selective inhibitors of parasitic GAPDH, investigators exploited differences near the cofactor-binding areas in parasitic GAPDH and hGAPDH. Several residues near the area where the adenosyl moiety of NAD binds in GBS GAPDH are different from the corresponding residues in hGAPDH (Fig 7). For example, in hGAPDH residues Pro36 and Phe37 form hydrophobic interactions with the adenine ring. In GBS GAPDH the corresponding loop is shorter since there is no residue matching Phe37, and Pro36 is replaced by a leucine residue. On the other side of the pocket Val101 in hGAPDH is substituted by Phe99 in GBS GAPH. In the extended pocket GBS GAPDH and hGAPDH exhibit several additional differences, which can be potentially exploited for designing inhibitors that bind tightly and selectively to GBS GAPD as compared to hGAPDH.

## Materials and Methods

### Crystallization, Data Collection and Processing

Wild type and mutant GBS GAPDH were purified as previously described [10]. The C152S mutant was generated using a standard protocol and purified as described for the wild type protein [10].

Wild type and mutant GBS GAPDH were purified as previously described [10]. Briefly, GBS GAPDH coding sequence was inserted into pET15b expression vector. The recombinant protein (carrying a 20-residue N-terminal tag containing hexa-histidine and a thrombin cleavage sequence) was expressed in *Escherichia coli* Rosetta (DE3) pLysS cells using 0.4 mM isopropyl thio-β-D-galactoside for induction at 22°C for 16 hrs. Recombinant GBS GAPDH was purified from soluble cellular extract by immobilized metal affinity chromatography on TALON (Clontech) resin followed by size exclusion chromatography on Superdex 200 column (GE Healthcare) in 25 mM Hepes buffer containing 100 mM NaCl, 5 mM β-mercaptoethanol, pH 7.35. GBS GAPDH eluted as a single peak of approximately 160 kDa molecular mass (expected molecular mass for a tetramer ~156 kDa calculated for the protein plus his-tag). The peak fractions were pooled and concentrated to a final concentration of 15 mg/ml and stored in small aliquots at -84°C. The purified protein was examined for enzyme activity.

For preparation of binary and ternary complexes the protein was incubated with 2 mM NAD or 2 mM NAD and 11 mM DL-G3H, respectively. DL-G3H (Sigma) contained a mixture of D- and L-G3H with 50% of the D-isomer. No cofactor was added for preparation of the apo-form or mixed-holo form crystals. Crystals were grown in hanging drop vapor diffusion at 295 K [10]. Quality of crystals were improved by using microseeding technique for which initial crystals were crushed into the reservoir solution and the suspension was added to the drops containing fresh protein and reservoir solution. Reservoir solutions for growing crystals used for structure determination contained 20–28% PEG4000, 0.1 M MES buffer, pH 6.5. For data collection crystals were transferred serially into reservoir solutions supplemented with 5, 10, 15 and 20% of glycerol (v/v) and then flash-frozen in liquid nitrogen.

Datasets for the apo enzyme, the mixed-holo complex and the new holo complex were collected on NE-CAT beamline 24-ID-C at the Advanced Photon Source (APS), Argonne, USA. Beamline 24-ID-C is equipped with a Dectris Pilatus 6M-F CCD detector. These datasets were processed with XDS [24,25] and SCALA [26] in the CCP4 suite [27] as part of the RAPD data-collection strategy at NE-CAT (https://rapd.chem.cornell.edu/rapd). Data for the ternary complex were collected on beamline BL12-2 at SSRL using an ADSC Q315 CCD detector. This dataset was processed with HKL-2000 [28].

### Structure Determination and Refinement

We used either a monomer or the entire tetramer (if isomorphous) of the *wt*GBS GAPDH structure [*4QX6*] as search model for molecular replacement. Crystal structures were solved with PHASER [29] in the CCP4 suite. After initial refinement, cofactor NAD^+^ could be unambiguously placed in the electron density in each of the four subunits for the holo and the ternary complexes, and in two subunits in the mixed-holo complex. For the ternary complex we also added the substrate D-G3H. After refinement of protein residues and ligands, water molecules were modeled into a difference electron density map (3σ contour level) using Coot [30]. In early refinement stages we used automatically generated NCS restraints but in the final stages GAPDH molecules were treated independently. Refinement and model building were performed with PHENIX [31–32], REFMAC5 [33] and Coot.

Structure validation was accomplished using PHENIX, MolProbity [22,34], QualityCheck (http://smb.slac.stanford.edu/jcsg/QC/) [35], and the new wwPDB X-ray validation server (http://wwpdb-validation.wwpdb.org/validservice/). PHENIX and REFMAC were used for final map calculations.

Data collection and refinement statistics are listed in Table 1. Final atomic coordinates and structure factors for the GBS GAPDH complexes have been deposited in PDB (entries *5JYF*, *5JYE*, *5JY6*, and *5JYA*).

All figures were prepared using PyMOL [36].

## Supporting Information

S1 Fig Holo GBS GAPDH complex (*5JY6*): electron-density map for cofactor NAD^+^. The figure displays the 2mFo-DFc electron density map (1σ contour level) for cofactor NAD^+^ in the active sites of the four subunits A-D of GBS GAPDH holo enzyme complex (*5JY6*). The NAD^+^ molecules are shown as stick models (C salmon, O red, N blue, P orange) and the neighboring protein residues as line models (C green, O red, N blue). The view is clipped at 8 Å.
(DOCX)

S2 Fig Ternary GBS GAPDH complex (*5JYA*): electron-density map for cofactor NAD^+^ and substrate D-G3H. The figure displays the 2mFo-DFc electron density map (1σ contour level) for cofactor NAD^+^ and substrate D-G3H in the active sites of the four subunits A-D of the GBS GAPDH ternary enzyme complex [*5JYA*]. The NAD^+^ and D-G3H molecules are shown as stick models (NAD^+^: C salmon, O red, N blue, P orange; D-G3H: C white, O red, P orange) and the neighboring protein residues as line models (C green, O red, N blue). The view is clipped at 8 Å.
(DOCX)

S3 Fig Ternary GBS GAPDH complex [*5JYA*]: electron-density omit map for substrate D-G3H. The figure displays the mFo-DFc electron density omit map (3σ contour level) for substrate G3H in the active sites of the four subunits A-D of the GBS GAPDH ternary enzyme complex. After removal of the substrate molecules the structure was refined by 10 cycles of maximum likelihood in REFMAC. The G3H molecules are shown as stick models (C white, O red, P orange) and the neighboring protein residues as line models (C green, O red, N blue). The view is clipped at 8 Å.
(DOCX)

S1 Table Listing of interfaces in the GBS GAPDH crystal structures. (DOCX)

S2 Table Listing of stable assemblies in the GBS GAPDH crystal structures. (DOCX)

S3 Table Root-mean-square deviations (*rmsd*) of individual subunits in GBS GAPDH structures. (DOCX)

S4 Table List of H-bonds involving NAD^+^ and D-G3H. (DOCX)

S5 Table Structure-based multiple sequence alignment by PROMALS3D of eukaryotic, prokaryotic and parasitic GAPDH. (DOCX)
