# Supplementary material for: Crystal Structures of Group B Streptococcus Glyceraldehyde-3-Phosphate Dehydrogenase: Apo-Form, Binary and Ternary Complexes
Source: PLoS One. 2016 Nov 22;11(11):e0165917. doi: 10.1371/journal.pone.0165917 (PMC5119734; doi:10.1371/journal.pone.0165917)
Supplement: S1 Fig — The figure displays the 2mFo-DFc electron density map (1σ contour level) for cofactor NAD+ in the active sites of the four subunits A-D of GBS GAPDH holo enzyme complex (5JY6). The NAD+ molecules are shown as stick models (C salmon, O red, N blue, P orange) and the neighboring protein residues as line models (C green, O red, N blue). The view is clipped at 8 Å. (DOCX) [file pone.0165917.s001.docx]

**S1 Fig. Holo GBS GAPDH complex (*5JY6*): electron-density map for cofactor NAD^+^.** The figure displays the 2mFo-DFc electron density map (1σ contour level) for cofactor NAD^+^ in the active sites of the four subunits A-D of GBS GAPDH holo enzyme complex (*5JY6*). The NAD^+^ molecules are shown as stick models (C salmon, O red, N blue, P orange) and the neighboring protein residues as line models (C green, O red, N blue). The view is clipped at 8 Å.

**
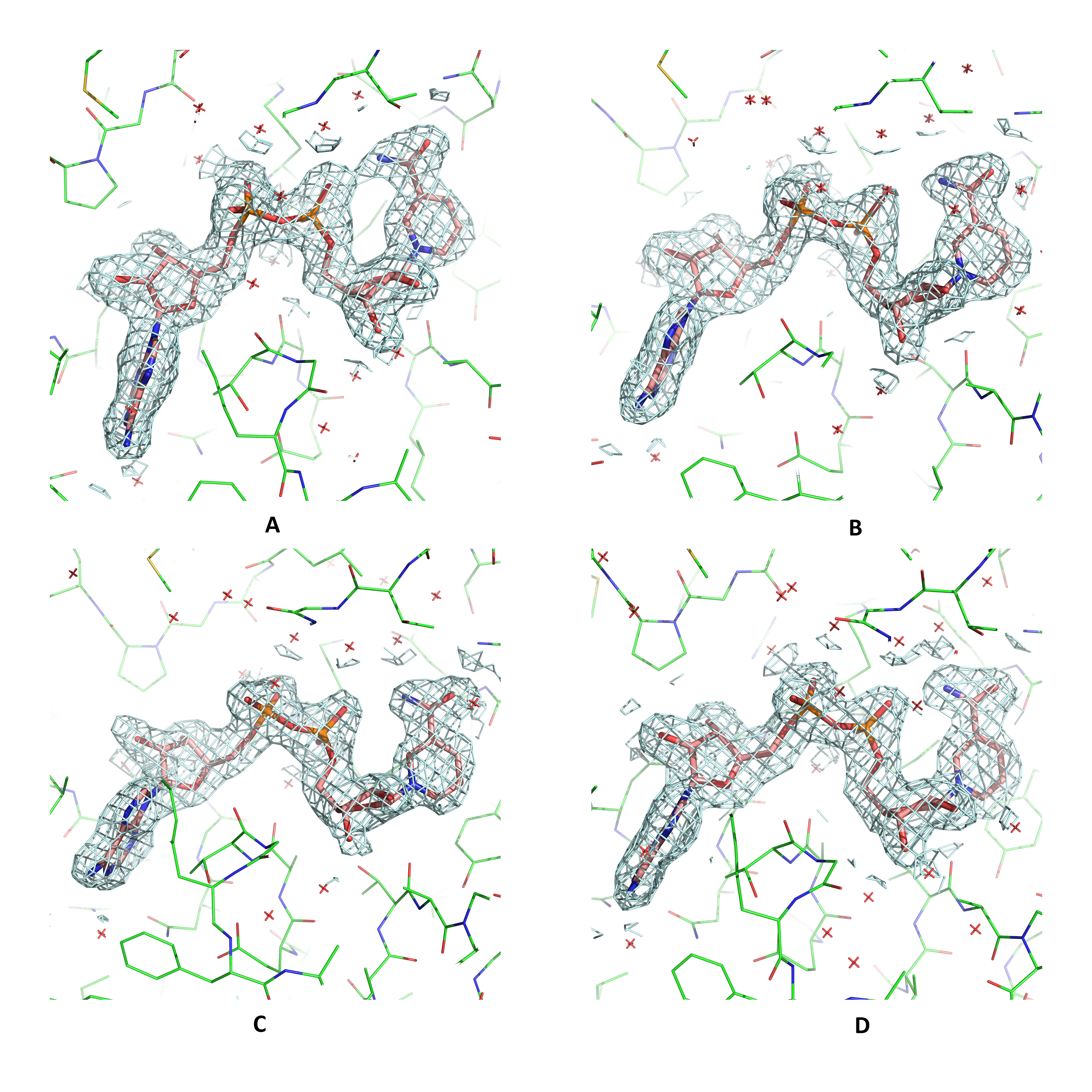
**
