# Supplementary material for: Crystal Structures of Group B Streptococcus Glyceraldehyde-3-Phosphate Dehydrogenase: Apo-Form, Binary and Ternary Complexes
Source: PLoS One. 2016 Nov 22;11(11):e0165917. doi: 10.1371/journal.pone.0165917 (PMC5119734; doi:10.1371/journal.pone.0165917)

**S2 Fig. Ternary GBS GAPDH complex (*5JYA*): electron-density map for cofactor NAD^+^ and substrate D-G3H.** The figure displays the 2mFo-DFc electron density map (1σ contour level) for cofactor NAD^+^ and substrate D-G3H in the active sites of the four subunits A-D of the GBS GAPDH ternary enzyme complex [*5JYA*]. The NAD^+^ and D-G3H molecules are shown as stick models (NAD^+^: C salmon, O red, N blue, P orange; D-G3H: C white, O red, P orange) and the neighboring protein residues as line models (C green, O red, N blue). The view is clipped at 8 Å.


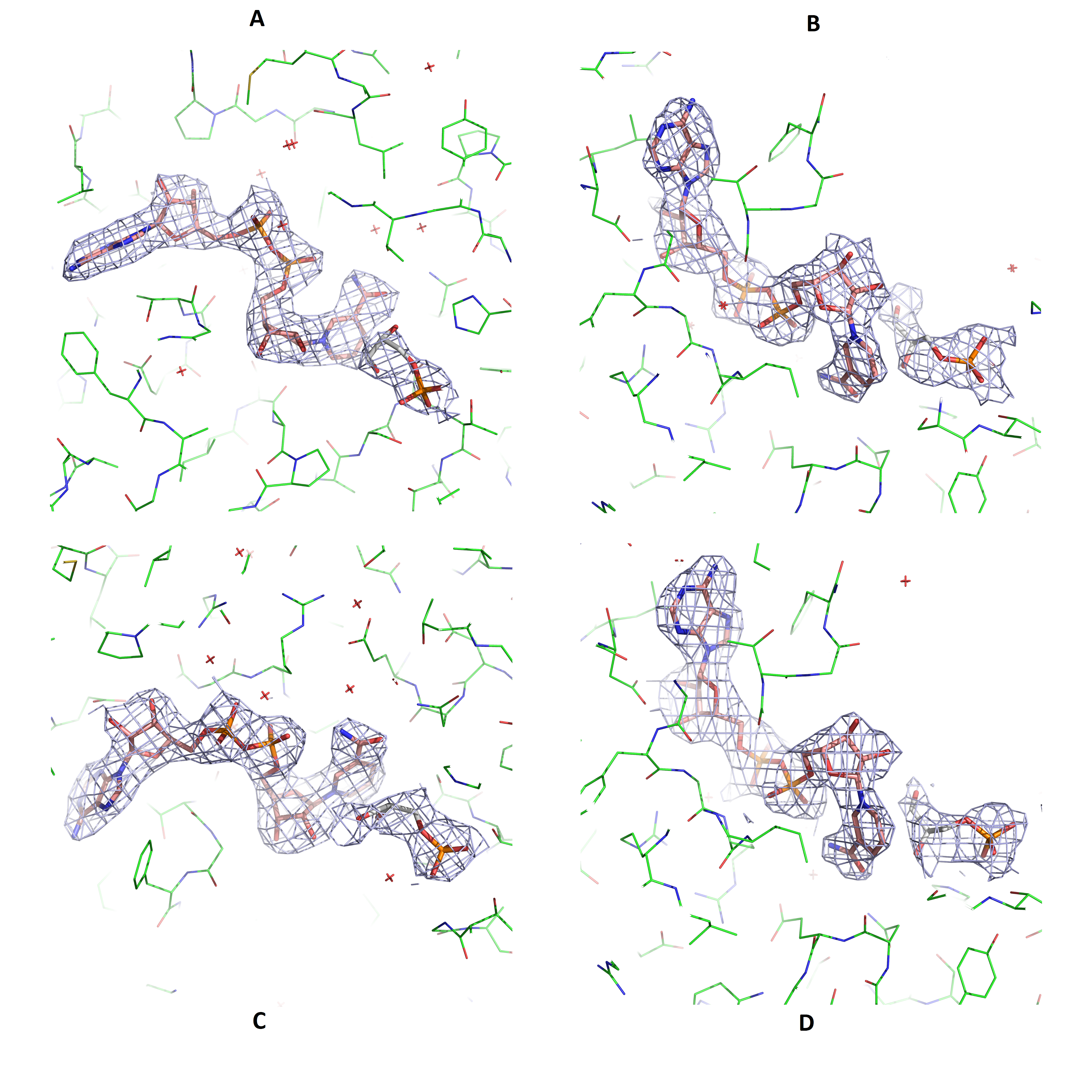

Supplement: S2 Fig — The figure displays the 2mFo-DFc electron density map (1σ contour level) for cofactor NAD+ and substrate D-G3H in the active sites of the four subunits A-D of the GBS GAPDH ternary enzyme complex [5JYA]. The NAD+ and D-G3H molecules are shown as stick models (NAD+: C salmon, O red, N blue, P orange; D-G3H: C white, O red, P orange) and the neighboring protein residues as line models (C green, O red, N blue). The view is clipped at 8 Å. (DOCX) [file pone.0165917.s002.docx]
