# Supplementary material for: Crystal Structures of Group B Streptococcus Glyceraldehyde-3-Phosphate Dehydrogenase: Apo-Form, Binary and Ternary Complexes
Source: PLoS One. 2016 Nov 22;11(11):e0165917. doi: 10.1371/journal.pone.0165917 (PMC5119734; doi:10.1371/journal.pone.0165917)

**S3 Fig. Ternary GBS GAPDH complex [*5JYA*]: electron-density omit map for substrate D-G3H.** The figure displays the mFo-DFc electron density omit map (3σ contour level) for substrate G3H in the active sites of the four subunits A-D of the GBS GAPDH ternary enzyme complex. After removal of the substrate molecules the structure was refined by 10 cycles of maximum likelihood in REFMAC. The G3H molecules are shown as stick models (C white, O red, P orange) and the neighboring protein residues as line models (C green, O red, N blue). The view is clipped at 8 Å.


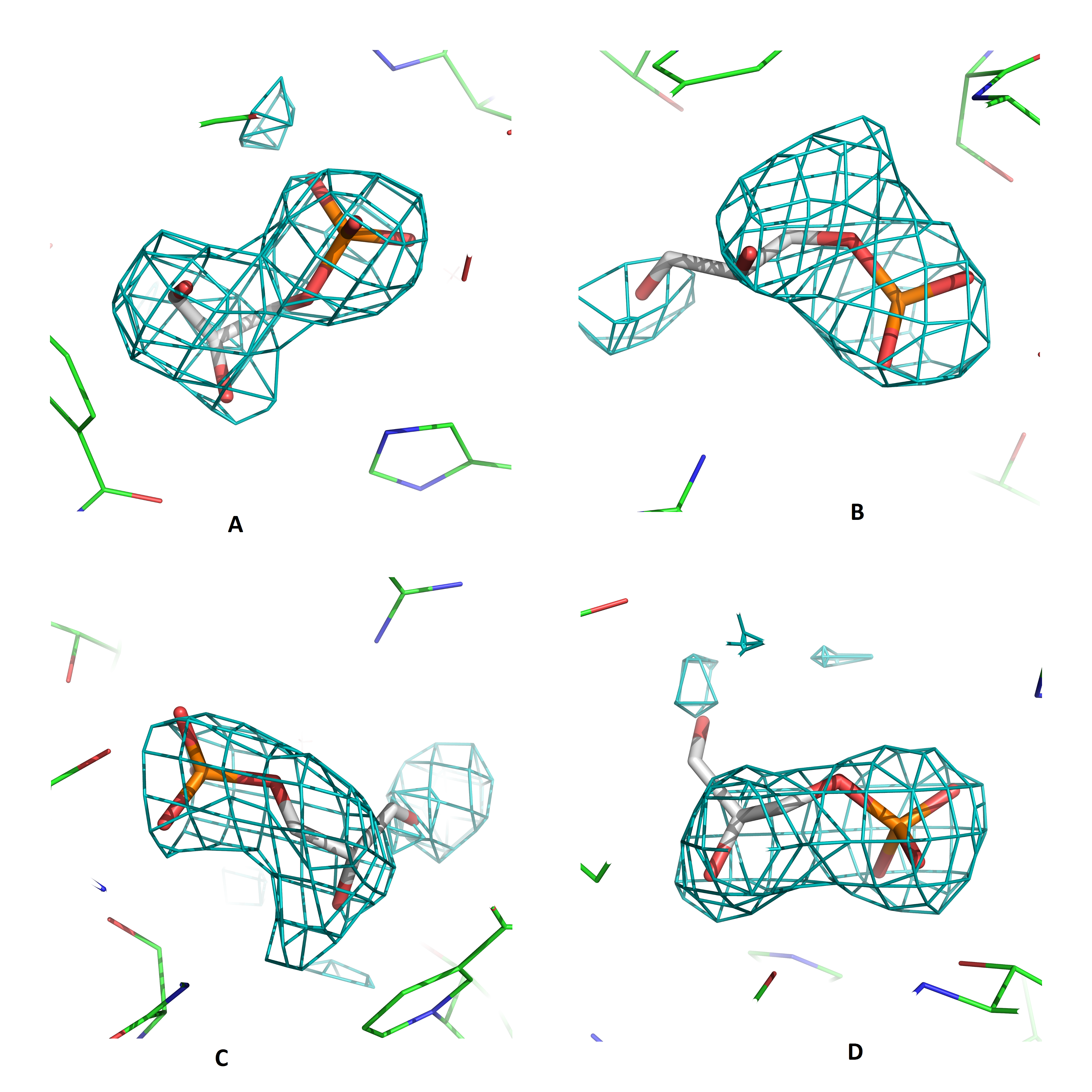

Supplement: S3 Fig — The figure displays the mFo-DFc electron density omit map (3σ contour level) for substrate G3H in the active sites of the four subunits A-D of the GBS GAPDH ternary enzyme complex. After removal of the substrate molecules the structure was refined by 10 cycles of maximum likelihood in REFMAC. The G3H molecules are shown as stick models (C white, O red, P orange) and the neighboring protein residues as line models (C green, O red, N blue). The view is clipped at 8 Å. (DOCX) [file pone.0165917.s003.docx]
