# Supplementary material for: Crystal Structures of Group B Streptococcus Glyceraldehyde-3-Phosphate Dehydrogenase: Apo-Form, Binary and Ternary Complexes
Source: PLoS One. 2016 Nov 22;11(11):e0165917. doi: 10.1371/journal.pone.0165917 (PMC5119734; doi:10.1371/journal.pone.0165917)
Supplement: S1 Table — (DOCX) [file pone.0165917.s004.docx]

**S1 Table. Listing of interfaces in the GBS GAPDH crystal structures.**

**Interface areas**

1. Apo (*5JYF*): interface subunits ^1^BSA [Å^2^] residues ^2^HB ^3^SB

P AB 3860 52+55=109 44 12

P CD 3856 52+56=108 45 12

R AC 2518 38+37=75 11 0

R BD 2634 40+40=80 11 1

Q AD 1093 17+17=34 9 4

Q BC 1068 17+17=34 9 4

1. Apo/Holo (*5JYE*): interface subunits ^1^BSA [Å^2^] residues ^2^HB ^3^SB

P AB 3832 54+52=106 39 12

P CD 3836 53+53=106 40 11

R AC 2674 40+40=80 11 0

R BD 2621 39+40=79 11 0

Q AD 1069 17+17=34 9 4

Q BC 1073 17+17=34 12 4

1. Holo (*5JY6*): interface subunits ^1^BSA [Å^2^] residues ^2^HB ^3^SB

P AB 3822 54+55=109 42 14

P CD 3795 52+53=105 42 14

R AC 2686 40+40=80 10 0

R BD 2690 40+40=79 10 0

Q AD 1065 16+16=32 9 4

Q BC 1050 16+16=32 9 4

1. Ternary (*5JYA*): interface subunits ^1^BSA [Å^2^] residues ^2^HB ^3^SB

P AB 3872 52+54=106 49 10

P CD 3893 55+54=109 49 10

R AC 2752 42+42=84 12 0

R BD 2790 42+44=86 11 0

Q AD 997 17+17=34 6 4

Q BC 1014 17+16=33 9 4

The listed interfaces are based on analysis with PDBePISA (<http://www.ebi.ac.uk/pdbe/pisa/>).

^1^BSA= buried surface area

^2^HB=hydrogen bonds

^3^SB= salt bridges

**Interface residues**

Interface Subunits Residues

P AB K171, Q172, G173, L174, M175, T176, I178, H179, L197, R198,

R201, A204, A205, N206, I207, V208, P209, G227, K228, L229,

D230, G231, A232, A233, Q234, V236, P237, V238, G241, S242,

V243, E245, V247, A248, T249, P277, I278, V279, S280, S281,

D282, D293, T295, Q296, K298, Q300, V302, D303, N305, Q306,

L307, V308, K309, V311, W313

R AC F10, G11, R12, R15, D34, L35, T36, N39, M40, L41, H43, L44, Y47, D48, T49, T50, Y181, T182, G183, Q185, M186, I187, L188, D189, G190, P191, H192, G195, D196, L197, R198, A200, R201, A202, G203, A204, A205, N206, P239, E317

Q AD H43, K46, Y47, D48, T49, R53, D276, P277, I278, V279, S281, D282,
