# Supplementary material for: Crystal Structures of Group B Streptococcus Glyceraldehyde-3-Phosphate Dehydrogenase: Apo-Form, Binary and Ternary Complexes
Source: PLoS One. 2016 Nov 22;11(11):e0165917. doi: 10.1371/journal.pone.0165917 (PMC5119734; doi:10.1371/journal.pone.0165917)
Supplement: S2 Table — (DOCX) [file pone.0165917.s005.docx]

**S2 Table. Listing of stable assemblies in the GBS GAPDH crystal structures.**

1. Apo (*5JYF*): subunits ^1^BSA [Å^2^]

ABCD 15030 (33% of ASA)

AB 3860 (15% of ASA)

CD 3860 (15% of ASA)

AC 2520 (9% of ASA)

BD 2630 (10% of ASA)

1. Apo/Holo (*5JYE*): subunits ^1^BSA [Å^2^]

ABCD 17640 (39% of ASA)

AB 4990 (19% of ASA)

CD 5000 (19% of ASA)

AC 5210 (19% of ASA)

BD 2620 (9% of ASA)

1. Holo (*5JY6*): subunits ^1^BSA [Å^2^]

ABCD 20210 (46% of ASA)

AB 6160 (24% of ASA)

CD 6130 (24% of ASA)

AC 5230 (20% of ASA)

BD 5240 (20% of ASA)

1. Ternary (*5JYA*): subunits ^1^BSA [Å^2^]

ABCD 21870 (50% of ASA)

AB 6940 (27% of ASA)

CD 6950 (27% of ASA)

AC 6020 (23% of ASA)

BD 6070 (22% of ASA)

The assemblies listed as stable based on PDBePISA (<http://www.ebi.ac.uk/pdbe/pisa/>) include ligands (NAD^+^, Mg^+2^, D-G3H) if applicable (Apo/Holo: NAD^+^, Mg^+2^ in subunits A and C; Holo: NAD^+^, Mg^+2^ in subunits A-D; Ternary: NAD^+^, D-G3H in subunits A-D).

^1^BSA= buried surface area

^2^ASA= accessible surface area
