# Supplementary material for: Crystal Structures of Group B Streptococcus Glyceraldehyde-3-Phosphate Dehydrogenase: Apo-Form, Binary and Ternary Complexes
Source: PLoS One. 2016 Nov 22;11(11):e0165917. doi: 10.1371/journal.pone.0165917 (PMC5119734; doi:10.1371/journal.pone.0165917)
Supplement: S3 Table — (DOCX) [file pone.0165917.s006.docx]

**S3 Table. Root-mean-square deviations (*rmsd*) of individual subunits in GBS GAPDH structures.**

1. Apo (4): *rmsd* 0.42-0.99Å (P interface dimers: subunits AB and CD)

AB *rmsd* 0.73Å (285 Cα atoms); AC *rmsd* 0.56Å (323 Cα atoms); AD *rmsd* 0.65Å (312 Cα atoms); BC *rmsd* 0.93Å (292 Cα atoms); BD *rmsd* 0.42Å (291 Cα atoms); CD *rmsd* 0.99Å (317 Cα atoms)

1. Holo (2)/Apo (2): *rmsd* 0.20-1.36Å (P interface dimers: subunits AB and CD)

AC *rmsd* 0.20Å (334 Cα atoms); AB *rmsd* 1.32Å (330 Cα atoms); AD *rmsd* 1.36Å (328 Cα atoms); BC *rmsd* 1.23Å (332 Cα atoms); BD *rmsd* 0.33Å (334 Cα atoms); CD *rmsd* 1.26Å (330 Cα atoms)

1. Holo (4): *rmsd* 0.06-0.14Å (P interface dimers subunits: AB and CD)

AB *rmsd* 0.14Å (333 Cα atoms); AC *rmsd* 0.11Å (330 Cα atoms); AD *rmsd* 0.09Å (332 Cα atoms); BC *rmsd* 0.12Å (329 Cα atoms); BD *rmsd* 0.13Å (332 Cα atoms); CD *rmsd* 0.06Å (329 Cα atoms)

1. Ternary (4): *rmsd* 0.09-0.26Å (P interface dimers: subunits AB and CD)

AC *rmsd* 0.26Å (330 Cα atoms); AB *rmsd* 0.14Å (330 Cα atoms); AD *rmsd* 0.16Å (330 Cα atoms); BC *rmsd* 0.11Å (331 Cα atoms); BD *rmsd* 0.09Å (332 Cα atoms); CD *rmsd* 0.11Å (333 Cα atoms)

**Rmsd values are calculated using CCP4 suite [28].**
