# Supplementary material for: Crystal Structures of Group B Streptococcus Glyceraldehyde-3-Phosphate Dehydrogenase: Apo-Form, Binary and Ternary Complexes
Source: PLoS One. 2016 Nov 22;11(11):e0165917. doi: 10.1371/journal.pone.0165917 (PMC5119734; doi:10.1371/journal.pone.0165917)
Supplement: S4 Table — (DOCX) [file pone.0165917.s007.docx]

**S4 Table. List of H-bonds involving NAD^+^ and D-G3H.**

**Apo/Holo Complex (*5JYE*)**

Subunit A

| 1 | A:NAD 401[ N1A] | 3.81 | A:GLU  77[ O  ] |
| --- | --- | --- | --- |
| 2 | A:NAD 401[ N6A] | 3.10 | A:ARG  78[ O  ] |
| 3 | A:NAD 401[ O7N] | 2.99 | A:ASN 316[ ND2] |
| 4 | A:NAD 401[ O4D] | 3.11 | A:THR 121[ OG1] |
| 5 | A:NAD 401[ O2N] | 3.03 | A:ARG  12[ N  ] |
| 6 | A:NAD 401[ O2N] | 2.90 | A:ILE  13[ N  ] |
| 7 | A:NAD 401[ O2A] | 2.99 | A:ARG  12[ N  ] |
| 8 | A:NAD 401[ O3B] | 3.87 | A:PHE  10[ N  ] |
|  |  |  |  |
|  |  |  |  |

Subunit C

| 1 | C:NAD 401[ N1A] | 3.82 | C:GLU  77[ O  ] |
| --- | --- | --- | --- |
| 2 | C:NAD 401[ N6A] | 2.87 | C:ARG  78[ O  ] |
| 3 | C:NAD 401[ O7N] | 2.99 | C:ASN 316[ ND2] |
| 4 | C:NAD 401[ O4D] | 3.12 | C:THR 121[ OG1] |
| 5 | C:NAD 401[ O2N] | 2.94 | C:ARG  12[ N  ] |
| 6 | C:NAD 401[ O2N] | 2.95 | C:ILE  13[ N  ] |
| 7 | C:NAD 401[ O2A] | 2.85 | C:ARG  12[ N  ] |

Subunit A

/A/ 509(HOH) / O [ O ]: /A/ 401(NAD) / N7N[ N ]: 3.49

/A/ 524(HOH) / O [ O ]: /A/ 401(NAD) / O3D[ O ]: 2.90

/A/ 509(HOH) / O [ O ]: /A/ 401(NAD) / O1N[ O ]: 2.90

/A/ 506(HOH) / O [ O ]: /A/ 401(NAD) / O2N[ O ]: 2.76

/A/ 516(HOH) / O [ O ]: /A/ 401(NAD) / O3 [ O ]: 3.42

/A/ 401(NAD) / O1A[ O ]: 2.45

/A/ 506(HOH) / O [ O ]: /A/ 401(NAD) / O5B[ O ]: 3.62

/A/ 520(HOH) / O [ O ]: /A/ 401(NAD) / N1A[ N ]: 2.96

/A/ 520(HOH) / O [ O ]: /A/ 401(NAD) / N6A[ N ]: 3.78

Subunit C

/C/ 505(HOH) / O [ O ]: /C/ 401(NAD) / N7N[ N ]: 3.64

/C/ 535(HOH) / O [ O ]: /C/ 401(NAD) / N1N[ N ]: 3.86

/C/ 505(HOH) / O [ O ]: /C/ 401(NAD) / O1N[ O ]: 2.99

/C/ 517(HOH) / O [ O ]: /C/ 401(NAD) / O2N[ O ]: 2.90

/C/ 517(HOH) / O [ O ]: /C/ 401(NAD) / O5B[ O ]: 3.62

/C/ 532(HOH) / O [ O ]: /C/ 401(NAD) / N1A[ N ]: 2.92

/C/ 532(HOH) / O [ O ]: /C/ 401(NAD) / N6A[ N ]: 3.70

**Holo (Binary) Complex (*5JY6*)**

Subunit A

| 1 | A:NAD 401[ N1A] | 3.74 | A:GLU  77[ O  ] |
| --- | --- | --- | --- |
| 2 | A:NAD 401[ N6A] | 2.92 | A:ARG  78[ O  ] |
| 3 | A:NAD 401[ O7N] | 2.96 | A:ASN 316[ ND2] |
| 4 | A:NAD 401[ O4D] | 3.16 | A:THR 121[ OG1] |
| 5 | A:NAD 401[ O2N] | 3.04 | A:ARG  12[ N  ] |
| 6 | A:NAD 401[ O2N] | 3.03 | A:ILE  13[ N  ] |
| 7 | A:NAD 401[ O2A] | 3.08 | A:ARG  12[ N  ] |

Subunit B

| 1 | B:NAD 401[ N1A] | 3.79 | B:GLU  77[ O  ] |
| --- | --- | --- | --- |
| 2 | B:NAD 401[ N6A] | 2.96 | B:ARG  78[ O  ] |
| 3 | B:NAD 401[ O7N] | 2.93 | B:ASN 316[ ND2] |
| 4 | B:NAD 401[ O4D] | 3.04 | B:THR 121[ OG1] |
| 5 | B:NAD 401[ O2N] | 2.97 | B:ARG  12[ N  ] |
| 6 | B:NAD 401[ O2N] | 2.99 | B:ILE  13[ N  ] |
| 7 | B:NAD 401[ O2A] | 3.00 | B:ARG  12[ N  ] |

Subunit C

| 1 | C:NAD 401[ N1A] | 3.89 | C:GLU  77[ O  ] |
| --- | --- | --- | --- |
| 2 | C:NAD 401[ N6A] | 3.08 | C:ARG  78[ O  ] |
| 3 | C:NAD 401[ O7N] | 2.84 | C:ASN 316[ ND2] |
| 4 | C:NAD 401[ O2N] | 3.00 | C:ARG  12[ N  ] |
| 5 | C:NAD 401[ O2N] | 3.04 | C:ILE  13[ N  ] |
| 6 | C:NAD 401[ O2A] | 3.06 | C:ARG  12[ N  ] |

Subunit D

| 1 | D:NAD 401[ N1A] | 3.78 | D:GLU  77[ O  ] |
| --- | --- | --- | --- |
| 2 | D:NAD 401[ N6A] | 3.01 | D:ARG  78[ O  ] |
| 3 | D:NAD 401[ O7N] | 2.92 | D:ASN 316[ ND2] |
| 4 | D:NAD 401[ O4D] | 3.04 | D:THR 121[ OG1] |
| 5 | D:NAD 401[ O2N] | 2.99 | D:ARG  12[ N  ] |
| 6 | D:NAD 401[ O2N] | 2.96 | D:ILE  13[ N  ] |
| 7 | D:NAD 401[ O2A] | 3.06 | D:ARG  12[ N  ] |

Subunit A

/A/ 516(HOH) / O [ O ]: /A/ 401(NAD) / O7N[ O ]: 3.71

/A/ 502(HOH) / O [ O ]: /A/ 401(NAD) / N7N[ N ]: 3.33

/A/ 516(HOH) / O [ O ]: /A/ 401(NAD) / N7N[ N ]: 2.94

/A/ 586(HOH) / O [ O ]: /A/ 401(NAD) / O2D[ O ]: 3.29

/A/ 586(HOH) / O [ O ]: /A/ 401(NAD) / O3D[ O ]: 2.60

/A/ 544(HOH) / O [ O ]: /A/ 401(NAD) / O3D[ O ]: 2.86

/A/ 502(HOH) / O [ O ]: /A/ 401(NAD) / O1N[ O ]: 2.79

/A/ 509(HOH) / O [ O ]: /A/ 401(NAD) / O2N[ O ]: 2.77

/A/ 556(HOH) / O [ O ]: /A/ 401(NAD) / O3 [ O ]: 3.29

/A/ 556(HOH) / O [ O ]: /A/ 401(NAD) / O1A[ O ]: 2.96

/A/ 573(HOH) / O [ O ]: /A/ 401(NAD) / O1A[ O ]: 3.78

/A/ 515(HOH) / O [ O ]: /A/ 401(NAD) / O1A[ O ]: 3.75

/A/ 515(HOH) / O [ O ]: /A/ 401(NAD) / O2A[ O ]: 2.77

/A/ 509(HOH) / O [ O ]: /A/ 401(NAD) / O5B[ O ]: 3.54

/A/ 592(HOH) / O [ O ]: /A/ 401(NAD) / N7A[ N ]: 2.87

/A/ 547(HOH) / O [ O ]: /A/ 401(NAD) / N1A[ N ]: 2.91

/A/ 592(HOH) / O [ O ]: /A/ 401(NAD) / N6A[ N ]: 3.49

/A/ 547(HOH) / O [ O ]: /A/ 401(NAD) / N6A[ N ]: 3.85

Subunit B

/B/ 509(HOH) / O [ O ]: /B/ 401(NAD) / O7N[ O ]: 3.73

/B/ 646(HOH) / O [ O ]: /B/ 401(NAD) / O7N[ O ]: 2.88

/B/ 509(HOH) / O [ O ]: /B/ 401(NAD) / N7N[ N ]: 2.91

/B/ 505(HOH) / O [ O ]: /B/ 401(NAD) / N7N[ N ]: 3.61

/B/ 634(HOH) / O [ O ]: /B/ 401(NAD) / N1N[ N ]: 3.59

/B/ 634(HOH) / O [ O ]: /B/ 401(NAD) / O2D[ O ]: 3.36

/B/ 560(HOH) / O [ O ]: /B/ 401(NAD) / O2D[ O ]: 3.32

/B/ 618(HOH) / O [ O ]: /B/ 401(NAD) / O2D[ O ]: 3.44

/B/ 531(HOH) / O [ O ]: /B/ 401(NAD) / O3D[ O ]: 2.84

/B/ 560(HOH) / O [ O ]: /B/ 401(NAD) / O3D[ O ]: 2.73

/B/ 569(HOH) / O [ O ]: /B/ 401(NAD) / O5D[ O ]: 3.80

/B/ 505(HOH) / O [ O ]: /B/ 401(NAD) / O1N[ O ]: 2.92

/B/ 569(HOH) / O [ O ]: /B/ 401(NAD) / O1N[ O ]: 2.98

/B/ 508(HOH) / O [ O ]: /B/ 401(NAD) / O2N[ O ]: 2.70

/B/ 569(HOH) / O [ O ]: /B/ 401(NAD) / O3 [ O ]: 3.78

/B/ 605(HOH) / O [ O ]: /B/ 401(NAD) / O3 [ O ]: 3.34

/B/ 512(HOH) / O [ O ]: /B/ 401(NAD) / O1A[ O ]: 3.64

/B/ 605(HOH) / O [ O ]: /B/ 401(NAD) / O1A[ O ]: 2.91

/B/ 512(HOH) / O [ O ]: /B/ 401(NAD) / O2A[ O ]: 2.78

/B/ 508(HOH) / O [ O ]: /B/ 401(NAD) / O5B[ O ]: 3.58

/B/ 620(HOH) / O [ O ]: /B/ 401(NAD) / N7A[ N ]: 2.94

/B/ 546(HOH) / O [ O ]: /B/ 401(NAD) / N1A[ N ]: 2.88

/B/ 546(HOH) / O [ O ]: /B/ 401(NAD) / N6A[ N ]: 3.94

/B/ 620(HOH) / O [ O ]: /B/ 401(NAD) / N6A[ N ]: 3.03

Subunit C

/C/ 506(HOH) / O [ O ]: /C/ 401(NAD) / O7N[ O ]: 3.82

/C/ 584(HOH) / O [ O ]: /C/ 401(NAD) / O7N[ O ]: 3.28

/C/ 506(HOH) / O [ O ]: /C/ 401(NAD) / N7N[ N ]: 2.89

/C/ 508(HOH) / O [ O ]: /C/ 401(NAD) / N7N[ N ]: 3.57

/C/ 536(HOH) / O [ O ]: /C/ 401(NAD) / O3D[ O ]: 2.77

/C/ 508(HOH) / O [ O ]: /C/ 401(NAD) / O1N[ O ]: 2.91

/C/ 517(HOH) / O [ O ]: /C/ 401(NAD) / O2N[ O ]: 2.86

/C/ 545(HOH) / O [ O ]: /C/ 401(NAD) / O3 [ O ]: 3.69

/C/ 545(HOH) / O [ O ]: /C/ 401(NAD) / O1A[ O ]: 3.05

/C/ 510(HOH) / O [ O ]: /C/ 401(NAD) / O1A[ O ]: 3.86

/C/ 510(HOH) / O [ O ]: /C/ 401(NAD) / O2A[ O ]: 2.89

/C/ 517(HOH) / O [ O ]: /C/ 401(NAD) / O5B[ O ]: 3.58

/C/ 578(HOH) / O [ O ]: /C/ 401(NAD) / N1A[ N ]: 2.90

Subunit D

/D/ 502(HOH) / O [ O ]: /D/ 401(NAD) / O7N[ O ]: 3.71

/D/ 504(HOH) / O [ O ]: /D/ 401(NAD) / N7N[ N ]: 3.35

/D/ 502(HOH) / O [ O ]: /D/ 401(NAD) / N7N[ N ]: 2.79

/D/ 608(HOH) / O [ O ]: /D/ 401(NAD) / N1N[ N ]: 3.45

/D/ 608(HOH) / O [ O ]: /D/ 401(NAD) / O2D[ O ]: 2.98

/D/ 587(HOH) / O [ O ]: /D/ 401(NAD) / O2D[ O ]: 3.26

/D/ 587(HOH) / O [ O ]: /D/ 401(NAD) / O3D[ O ]: 2.51

/D/ 520(HOH) / O [ O ]: /D/ 401(NAD) / O3D[ O ]: 3.02

/D/ 568(HOH) / O [ O ]: /D/ 401(NAD) / O5D[ O ]: 3.80

/D/ 504(HOH) / O [ O ]: /D/ 401(NAD) / O1N[ O ]: 2.79

/D/ 568(HOH) / O [ O ]: /D/ 401(NAD) / O1N[ O ]: 2.98

/D/ 507(HOH) / O [ O ]: /D/ 401(NAD) / O2N[ O ]: 2.84

/D/ 604(HOH) / O [ O ]: /D/ 401(NAD) / O3 [ O ]: 3.77

/D/ 510(HOH) / O [ O ]: /D/ 401(NAD) / O1A[ O ]: 3.79

/D/ 604(HOH) / O [ O ]: /D/ 401(NAD) / O1A[ O ]: 3.11

/D/ 510(HOH) / O [ O ]: /D/ 401(NAD) / O2A[ O ]: 2.82

/D/ 507(HOH) / O [ O ]: /D/ 401(NAD) / O5B[ O ]: 3.55

/D/ 595(HOH) / O [ O ]: /D/ 401(NAD) / N7A[ N ]: 3.00

/D/ 557(HOH) / O [ O ]: /D/ 401(NAD) / N1A[ N ]: 2.90

/D/ 595(HOH) / O [ O ]: /D/ 401(NAD) / N6A[ N ]: 3.58

/D/ 557(HOH) / O [ O ]: /D/ 401(NAD) / N6A[ N ]: 3.89

**Ternary Complex (5JYA)**

Subunit A

| 1 | A:NAD 401[ N1A] | 3.73 | A:GLU  77[ O  ] |
| --- | --- | --- | --- |
| 2 | A:NAD 401[ N6A] | 3.19 | A:ARG  78[ O  ] |
| 3 | A:NAD 401[ O7N] | 3.11 | A:ASN 316[ ND2] |
| 4 | A:NAD 401[ O4D] | 3.16 | A:THR 121[ OG1] |
| 5 | A:NAD 401[ O2N] | 2.83 | A:ARG  12[ N  ] |
| 6 | A:NAD 401[ O2N] | 2.98 | A:ILE  13[ N  ] |
| 7 | A:NAD 401[ O2A] | 3.09 | A:ARG  12[ N  ] |

| 1 | A:G3H 402[ O2 ] | 3.42 | A:SER 152[ OG ] |
| --- | --- | --- | --- |
| 2 | A:G3H 402[ O1 ] | 3.13 | A:SER 152[ N  ] |
| 3 | A:G3H 402[ O1 ] | 3.25 | A:SER 152[ OG ] |
| 4 | A:G3H 402[ O1P] | 3.49 | A:SER 152[ OG ] |
| 5 | A:G3H 402[ O1P] | 3.31 | A:HIS 179[ NE2] |
| 6 | A:G3H 402[ O2P] | 2.88 | A:SER 151[ OG ] |
| 7 | A:G3H 402[ O2P] | 3.83 | A:SER 152[ N  ] |
| 8 | A:G3H 402[ O2P] | 2.93 | A:THR 153[ N  ] |
| 9 | A:G3H 402[ O2P] | 3.76 | A:SER 152[ OG ] |
| 10 | A:G3H 402[ O3P] | 3.12 | A:THR 212[ OG1] |
| 11 | A:G3H 402[ O4P] | 3.10 | A:GLY 213[ N  ] |

Subunit B

| 1 | B:NAD 401[ N1A] | 3.61 | B:GLU  77[ O  ] |
| --- | --- | --- | --- |
| 2 | B:NAD 401[ N6A] | 2.97 | B:ARG  78[ O  ] |
| 3 | B:NAD 401[ O7N] | 3.01 | B:ASN 316[ ND2] |
| 4 | B:NAD 401[ O2N] | 3.05 | B:ARG  12[ N  ] |
| 5 | B:NAD 401[ O2N] | 3.13 | B:ILE  13[ N  ] |
| 6 | B:NAD 401[ O2A] | 2.95 | B:ARG  12[ N  ] |

| 1 | B:G3H 402[ O2 ] | 3.72 | B:SER 152[ OG ] |
| --- | --- | --- | --- |
| 2 | B:G3H 402[ O1 ] | 3.71 | B:ARG 235[ NH2] |
| 3 | B:G3H 402[ O1P] | 3.42 | B:SER 152[ OG ] |
| 4 | B:G3H 402[ O1P] | 3.81 | B:SER 152[ N  ] |
| 5 | B:G3H 402[ O3P] | 2.94 | B:GLY 213[ N  ] |
| 6 | B:G3H 402[ O3P] | 3.86 | B:THR 212[ OG1] |
| 7 | B:G3H 402[ O4P] | 2.85 | B:SER 151[ OG ] |
| 8 | B:G3H 402[ O4P] | 2.79 | B:THR 153[ N  ] |
| 9 | B:G3H 402[ O4P] | 3.63 | B:SER 152[ OG ] |
| 10 | B:G3H 402[ O4P] | 3.70 | B:SER 152[ N  ] |
|  |  |  |  |

Subunit C

| 1 | C:NAD 401[ N1A] | 3.66 | C:GLU  77[ O  ] |
| --- | --- | --- | --- |
| 2 | C:NAD 401[ N6A] | 3.10 | C:ARG  78[ O  ] |
| 3 | C:NAD 401[ O7N] | 3.06 | C:ASN 316[ ND2] |
| 4 | C:NAD 401[ O4D] | 3.12 | C:THR 121[ OG1] |
| 5 | C:NAD 401[ O2N] | 3.03 | C:ARG  12[ N  ] |
| 6 | C:NAD 401[ O2N] | 2.90 | C:ILE  13[ N  ] |
| 7 | C:NAD 401[ O2A] | 3.15 | C:ARG  12[ N  ] |

| 1 | C:G3H 402[ O2P] | 3.18 | C:THR 212[ OG1] |
| --- | --- | --- | --- |
| 2 | C:G3H 402[ O2P] | 2.53 | C:THR 153[ OG1] |
| 3 | C:G3H 402[ O3P] | 2.71 | C:GLY 213[ N  ] |
| 4 | C:G3H 402[ O4P] | 3.78 | C:SER 152[ OG ] |
| 5 | C:G3H 402[ O4P] | 2.70 | C:SER 151[ OG ] |
| 6 | C:G3H 402[ O4P] | 3.70 | C:SER 152[ N  ] |
| 7 | C:G3H 402[ O4P] | 2.97 | C:THR 153[ N  ] |

Subunit D

| 1 | D:NAD 401[ N1A] | 3.69 | D:GLU  77[ O  ] |
| --- | --- | --- | --- |
| 2 | D:NAD 401[ N6A] | 3.11 | D:ARG  78[ O  ] |
| 3 | D:NAD 401[ O7N] | 2.98 | D:ASN 316[ ND2] |
| 4 | D:NAD 401[ O4D] | 3.03 | D:THR 121[ OG1] |
| 5 | D:NAD 401[ O2N] | 2.89 | D:ARG  12[ N  ] |
| 6 | D:NAD 401[ O2N] | 2.96 | D:ILE  13[ N  ] |
| 7 | D:NAD 401[ O2A] | 3.21 | D:ARG  12[ N  ] |

| 1 | D:G3H 402[ O2 ] | 3.12 | D:NAD 401[ O7N] |
| --- | --- | --- | --- |

Subunit A

/A/ 501(HOH) / O [ O ]: /A/ 401(NAD) / O7N[ O ]: 3.94

/A/ 514(HOH) / O [ O ]: /A/ 401(NAD) / O2N[ O ]: 2.76

/A/ 527(HOH) / O [ O ]: /A/ 401(NAD) / O3 [ O ]: 3.69

/A/ 401(NAD) / O1A[ O ]: 2.84

/A/ 514(HOH) / O [ O ]: /A/ 401(NAD) / O5B[ O ]: 3.53

/A/ 511(HOH) / O [ O ]: /A/ 402(G3H) / O3P[ O ]: 3.86

/A/ 533(HOH) / O [ O ]: /A/ 402(G3H) / O3P[ O ]: 2.88

/A/ 511(HOH) / O [ O ]: /A/ 402(G3H) / O4P[ O ]: 3.02

Subunit B

/B/ 508(HOH) / O [ O ]: /B/ 401(NAD) / N7N[ N ]: 3.30

/B/ 536(HOH) / O [ O ]: /B/ 401(NAD) / O3D[ O ]: 3.03

/B/ 508(HOH) / O [ O ]: /B/ 401(NAD) / O1N[ O ]: 2.99

/B/ 501(HOH) / O [ O ]: /B/ 401(NAD) / O1N[ O ]: 3.73

/B/ 516(HOH) / O [ O ]: /B/ 401(NAD) / O2N[ O ]: 2.86

/B/ 516(HOH) / O [ O ]: /B/ 401(NAD) / O3 [ O ]: 3.96

/B/ 501(HOH) / O [ O ]: /B/ 401(NAD) / O1A[ O ]: 3.84

/B/ 401(NAD) / O2A[ O ]: 3.03

/B/ 516(HOH) / O [ O ]: /B/ 401(NAD) / O5B[ O ]: 3.41

Subunit C

/C/ 511(HOH) / O [ O ]: /C/ 401(NAD) / N7N[ N ]: 3.26

/C/ 501(HOH) / O [ O ]: /C/ 401(NAD) / O1N[ O ]: 3.60

/C/ 532(HOH) / O [ O ]: /C/ 401(NAD) / O2N[ O ]: 2.94

/C/ 532(HOH) / O [ O ]: /C/ 401(NAD) / O3 [ O ]: 3.98

/C/ 501(HOH) / O [ O ]: /C/ 401(NAD) / O1A[ O ]: 3.70

/C/ 501(HOH) / O [ O ]: /C/ 401(NAD) / O2A[ O ]: 2.87

/C/ 502(HOH) / O [ O ]: /C/ 401(NAD) / O2A[ O ]: 2.91

/C/ 532(HOH) / O [ O ]: /C/ 401(NAD) / O5B[ O ]: 3.86

/C/ 522(HOH) / O [ O ]: /C/ 402(G3H) / O1 [ O ]: 3.72

/C/ 522(HOH) / O [ O ]: /C/ 402(G3H) / O1P[ O ]: 3.51

/C/ 522(HOH) / O [ O ]: /C/ 402(G3H) / O3P[ O ]: 3.08

Subunit D

/D/ 516(HOH) / O [ O ]: /D/ 401(NAD) / O7N[ O ]: 3.88

/D/ 516(HOH) / O [ O ]: /D/ 401(NAD) / N7N[ N ]: 3.14

/D/ 502(HOH) / O [ O ]: /D/ 401(NAD) / N7N[ N ]: 3.33

/D/ 502(HOH) / O [ O ]: /D/ 401(NAD) / O1N[ O ]: 2.72

/D/ 539(HOH) / O [ O ]: /D/ 401(NAD) / O2A[ O ]: 2.82

/D/ 514(HOH) / O [ O ]: /D/ 401(NAD) / N1A[ N ]: 2.97
