# Supplementary material for: Crystal Structures of Group B Streptococcus Glyceraldehyde-3-Phosphate Dehydrogenase: Apo-Form, Binary and Ternary Complexes
Source: PLoS One. 2016 Nov 22;11(11):e0165917. doi: 10.1371/journal.pone.0165917 (PMC5119734; doi:10.1371/journal.pone.0165917)
Supplement: S5 Table — (DOCX) [file pone.0165917.s008.docx]

# S5 Table. Structure-based multiple sequence alignment by PROMALS3D of eukaryotic, prokaryotic and parasitic GAPDH

| 1U8F_O |  | 3 | KVKV**G**V**NGFGRIGR**LVT**R**AAFNSGKVDIVA**IND**PFI**D**LNYMVYMFQ**YD**STHGKFH**GTV**KAENGKLVI**NG**N | 72 |
| --- | --- | --- | --- | --- |
| 1DC6_A |  | 1 | TIKV**G**I**NGFGRIGR**IVF**R**AAQKRSDIEIVA**IND**-LL**D**ADYMAYMLK**YD**STHGRFD**GTV**EVKDGHLIV**NG**K | 69 |
| 1VSV_A |  | 2 | TATL**G**I**NGFGRIGR**LVL**R**ACMERNDITVVA**IND**PFM**D**VEYMAYLLK**YD**SVHGNFN**GTV**EVSGKDLCI**NG**K | 71 |
| 5JY6_A |  | 2 | VVKV**G**I**NGFGRIGR**LAF**R**RIQNVEGVEVTR**IND**-LT**D**PNMLAHLLK**YD**TTQGRFD**GTV**EVKEGGFEV**NG**Q | 70 |
| Consensus | ss: |  | eeeeee hhhhhhhhhhhh eeeee hhhhhhhhhh eeeee eeee e |  |

**Region 1**

| 1U8F_O |  | 73 | PITIFQERD**P**SKIK**W**GDA**G**AEY**V**V**E**S**TG**V**F**TTM**E**K**A**GA**H**LQ-G**GAK**R**V**IISA**P**SAD-APMF**V**MGV**N**HEKY | 140 |
| --- | --- | --- | --- | --- |
| 1DC6_A |  | 70 | KIRVTAERD**P**ANLK**W**DEV**G**VDV**V**A**E**A**TG**LFLTD**E**T**A**RK**H**IT-A**GAK**K**V**VMTG**P**SKDNTPMF**V**KGA**N**FDKY | 138 |
| 1VSV_A |  | 72 | VVKVFQAKD**P**AEIP**W**GAS**G**AQI**V**C**E**S**TG**V**F**TTE**E**K**A**SL**H**LK-G**GAK**K**V**IISA**P**PKDNVPMY**V**MGV**N**NTEY | 140 |
| 5JY6_A |  | 71 | FVKVSAERE**P**ANID**W**ATD**G**VEI**V**L**E**A**TG**F**F**ASK**E**K**A**EQ**H**IHEN**GAK**K**V**VITA**P**GGNDVKTV**V**FNT**N**HDIL | 140 |
| Consensus | ss: |  | eeeeee hhh hhh eeeee eeee hhhhhhhhh eeeee eeeeee hhh |  |

**Region 2**

| 1U8F_O |  | 141 | DNS-LKII**S**N**ASCTTNCLAP**L**AK**VIH**D**N**FG**IVE**GLMTT**V**H**AI**T**AT**Q**KTV**DGP**S--G-KLW**R**DG**R**GALQ**NI** | 206 |
| --- | --- | --- | --- | --- |
| 1DC6_A |  | 139 | AGQ--DIV**S**N**ASCTTNCLAP**L**AK**VIN**D**N**FG**IIE**GLMTT**V**H**AT**T**AT**Q**KTV**DGP**S--H-KDW**R**GG**R**GASQ**NI** | 203 |
| 1VSV_A |  | 141 | DPSKFNVI**S**N**ASCTTNCLAP**L**AK**IIN**D**K**FG**IVE**GLMTT**V**H**SL**T**AN**Q**LTV**DGP**SKGG-KDW**R**AG**R**CAGN**NI** | 209 |
| 5JY6_A |  | 141 | DGT-ETVI**S**G**ASCTTNCLA**PM**AK**ALQ**D**N**FG**VKQ**GLMTT**I**H**AY**T**GD**Q**MIL**DGP**H--RGGDL**R**RA**R**AGAA**NI** | 207 |
| Consensus | ss: |  | eeee hhhhhhhhhhhhhhhhhhhheeeeeee hhhhh ee |  |

| 1U8F_O |  | 207 | I**P**A**STGAAKA**V**G**K**V**I**P**E**LNGKL**T**G**M**A**F**RVP**TANV**SV**VD**L**TCR**L**E**K**PAKYDDIKKVV**K**Q**A**SEGPLKGIL**GY** | 276 |
| --- | --- | --- | --- | --- |
| 1DC6_A |  | 204 | I**P**S**STGAAKA**V**G**K**V**L**P**E**LNGKL**T**G**M**A**F**RVP**TPNV**SV**VD**L**TVR**L**E**K**AATYEQIKAAV**K**A**A**AEGEMKGVL**GY** | 273 |
| 1VSV_A |  | 210 | I**P**A**STGAAKA**V**G**K**V**I**P**A**LNGKL**T**G**M**A**I**RVP**TPDV**SV**VD**L**TCK**L**A**K**PASIEEIYQAV**K**E**A**SNGPMKGIM**GY** | 279 |
| 5JY6_A |  | 208 | V**P**N**STGAAKA**I**G**L**V**I**P**E**LNGKL**D**G**A**A**Q**RVP**VPTG**SV**TE**L**VAT**L**E**K**DVTVEEVNAAM**K**A**A**A----NDSY**GY** | 273 |
| Consensus | ss: |  | ee hhhhhhhhhhhhh eeee eeeeeeeeee hhhhhhhhhhhh ee |  |

| 1U8F_O |  | 277 | **T**EHQV**VS**S**D**FNSDTHS**S**T**FD**AGAGIALN---DHF**VK**LI**SWYDNE**FG**Y**SNRVVDLMAHMASKE- | 335 |
| --- | --- | --- | --- | --- |
| 1DC6_A |  | 274 | **T**EDDV**VS**T**D**FNGEVCT**S**V**FD**AKAGIALN---DNF**VK**LV**SWYDNE**TG**Y**SNKVLDLIAHISK--- | 330 |
| 1VSV_A |  | 280 | **T**SDDV**VS**T**D**FIGCKYS**S**I**FD**KNACIALN---DSF**VK**LI**SWYDNE**SG**Y**SNRLVDLAVYVASRGL | 339 |
| 5JY6_A |  | 274 | **T**EDPI**VS**S**D**IVGISYG**S**L**FD**ATQTKVQTVDGNQL**VK**VV**SWYDNE**MS**Y**TSQLVRTLEYFAKIA- | 335 |
| Consensus | ss: |  | eee eee hhhhhhee eeeeeeee hhhhhhhhhhhhhhhh |  |

**Region 3**

1U8F: human GAPDH; 1DC6: *Escherichia coli* GAPDH; 1VSV: *Cryptosporidium parvum* GAPDH; 5JYE: GBS GAPDH

Sequence identity to GBS GAPDH: *E. coli* (51%), *C. parvum* (46%), human (44%); identical residues in all four structures (118) in bold Consensus secondary structure: e = strand; h = helix

Regions 1-3 highlighted (in orange, green and red as in Figure 6) in GBS GAPDH. Underlined in GBS GAPDH are residues that interact with cofactor NAD.

PROMALS3D multiple sequence and structure alignment server (<http://prodata.swmed.edu/promals3d/)>
